# Evaluating Patient and Professional Satisfaction and Documentation Time Reduction Through AI-Driven Automatic Clinical Note Generation in Primary Care: Proof-of-Concept Study

**DOI:** 10.2196/80549

**Published:** 2026-03-24

**Authors:** Aïna Fuster-Casanovas, Josep Vidal-Alaball, Carlos Alonso, Queralt Miró Catalina, Daniel Hugo Heinisch, José Alberto Domínguez-Alonso, Gustavo Isaac Jurado Hamud, Ruthy Acosta-Rojas, Arlett Adriana Torres-Mercado, Jordi Ros Baró, Montserrat Ciurana Tebé, Alberto Castaño, Laia Sola Reguant, Anna Gomez-Fernandez

**Affiliations:** 1 Innovation Unit, Quality, Processes and Innovation Directorate, Vall d'Hebron University Hospital, Institut Català de la Salut, Barcelona, Spain; 2 eHealth Lab Research Group, School of Health Sciences and eHealth Centre, Universitat Oberta de Catalunya, Barcelona, Spain; 3 Research and Innovation Unit, Gerència d'Atenció Primària i a la Comunitat de la Catalunya Central, Institut Català de la Salut, Carrer Soler i March, 6, Manresa, 08242, Spain, 34 936930040, 34 936930040; 4 Health Promotion in Rural Areas Research Group, Institut Universitari d'Investigació en Atenció Primària Jordi Gol, Manresa, Spain; 5 Department of Medicine, Faculty of Medicine, Universitat de Vic - Universitat Central de Catalunya, Vic, Spain; 6 Recog Analytics, Madrid, Spain; 7 Centre d'Atenció Primària Vilafranca Nord, Gerència d'Atenció Primaria i a la Comuitat del Penedès, Institut Català de la Salut, Vilafranca del Penedès, Spain; 8 Disease, Cardiovascular Risk, and Lifestyle in Primary Health Care Research Group, Institut Universitari d'Investigació en Atenció Primària Jordi Gol, L’Hospitalet de Llobregat, Spain; 9 Research Group in Teaching in Primary Care., Institut Universitari d'Investigació en Atenció Primària Jordi Gol, Barcelona, Spain; 10 Centelles Primary Care Centre, Centelles, Spain; 11 Moià Primary Care Team, Gerència d'Atenció Primària i a la Comunitat de la Catalunya Central, Institut Català de la Salut, Moià, Spain; 12 Plaça Catalunya Primary Care Team, Gerència d'Atenció Primària i a la Comunitat de la Catalunya Central, Institut Català de la Salut, Manresa, Spain; 13 Center for the Integration of Medicine and Innovative Technologies, Fundación Leitat, Barcelona, Spain

**Keywords:** primary health care, patient satisfaction, artificial intelligence, medical records systems, computerized, patient-centered care

## Abstract

**Background:**

The workload that stems from writing clinical histories is one of the main sources of stress and overload for primary care professionals, accounting for up to 43% of the working day. The introduction of technology, specifically artificial intelligence (AI), in the field of health could significantly reduce the time spent writing clinical reports without compromising the quality of care.

**Objective:**

The objective of this study is to evaluate the impact of implementing an AI solution for the automatic transcription of consultations in several primary care centers in Catalonia.

**Methods:**

A proof of concept of a multicenter study was carried out with alternating assignment of consultations to the intervention group (use of an AI assistant that automatically generates consultation notes) or control group (usual clinical practice). The impact was evaluated through the recorded documentation time and the initial quality of the transcription measured with the Levenshtein distance expressed as corrected words per minute, complemented by a qualitative categorization of clinician-reported errors and the perceived satisfaction of patients and professionals through questionnaires evaluated through a Likert scale.

**Results:**

For the intervention group, the average processing time was 6.63%, while the review time by the professional amounted to 15.2%. Because documentation-time data were not available for the control group, no direct between-group comparison of time savings was possible; time-related findings are therefore exploratory and limited to intervention-group process and review metrics. Levenshtein-based estimates showed that in most cases, the review was <24 words per minute and 26% of drafts required no edits, indicating a high-quality initial transcription. A qualitative analysis of clinician feedback showed that context or meaning errors were the most frequent, while unsupported additions or hallucinations were uncommon. The satisfaction surveys were answered by 289 patients and 213 professionals. Patient satisfaction was high (≥4/5), with no statistically significant differences between the control and intervention groups. The professionals rated the audio quality at 9.06 out of 10 (SD 1.18; medicine) and 7.62 out of 10 (SD 1.58; nursing) and the transcription at 8.14 out of 10 (SD 1.74) and 6.93 out of 10 (SD 1.52), respectively.

**Conclusions:**

The implementation of an AI tool was feasible in routine primary care, was well accepted by clinicians, and did not negatively affect patient satisfaction, with a generally low transcription review burden. However, this proof-of-concept study does not allow conclusions about comparative time savings, and adequately powered randomized studies are needed to confirm benefits for care quality and efficiency.

## Introduction

Accurate clinical documentation is essential for monitoring and supporting clinical decision-making in primary care, but it is also one of the main sources of administrative burden for health care professionals. With the widespread adoption of electronic health records (EHRs), this burden has increased significantly: recent studies show that health care professionals can spend up to 43% of their health care activity time managing EHRs during outpatient visits [1]. This context has contributed to the increase in burnout among primary care professionals, as documented in several recent publications [2,3].

In response to this challenge, artificial intelligence (AI), especially automatic speech recognition (ASR) and natural language processing technologies, has emerged as a promising solution to automate the transcription and writing of clinical notes [4]. Several studies have shown that integrating ASR with large language models (LLMs), such as GPT-4, can generate draft clinical notes of a quality comparable to those prepared by health care professionals, improving efficiency and reducing transcription errors [5].

In addition, the use of AI tools that automatically record and transcribe consultations has shown a significant reduction in the time spent on documentation and an improvement in the satisfaction of both professionals and patients. For example, a recent study showed that the use of these tools can reduce documentation time by 20% and after-hours work by 30% [6].

Despite these advances and the fact that some studies have already been carried out in the field of primary care, most of the research is based on simulated electronic records and mainly in the English language, which differs from everyday clinical practice in non-English-speaking settings [7,8]. In Catalonia, where primary care plays a fundamental role in the health care system, it is essential to evaluate the effectiveness of these technologies in real, multilingual contexts with specific privacy and regulatory compliance requirements.

In this context, the project evaluated a proof of concept of an AI-powered solution (Relisten) developed by Recog Analytics for the automatic transcription and structuring of consultations in several Primary Care Centers (CAPs) in Catalonia. The study had the following main aims: (1) evaluate the satisfaction perceived by patients with the care received, (2) measure the satisfaction of professionals with the quality of the notes generated and the dynamics of the consultation, and (3) quantify the savings in time spent on clinical documentation.

This article presents the results of this study, providing evidence on the real applicability of generative AI in everyday primary care, in a specific linguistic and health care context such as that of Catalonia.

## Methods

### Design

A proof-of-concept multicenter study was conducted in which primary care nursing and medical professionals used an AI-powered tool during consultations (with the patient’s written informed consent).

### Study Population

The study population was made up of people who came in person to visit the CAPs of the public health system of Catalonia. Both unscheduled visits (emergencies) and first visits and follow-up visits for chronic diseases were included, as long as the responsible professionals also participated in the study. The CAPs that participated were CAP Amposta, CAP Artés, CAP Centelles, CAP Moià, CAP Plaça Catalunya (Manresa), CAP Sallent, CAP Súria, CAP Vilafranca Nord, and the Barcelona Esquerra Primary Care Health Consortium.

Given the nature of the tool and its applicability to most clinical practice settings, no prior selection of patients was made, and therefore, everyone who came to the center and who met the inclusion criteria was invited to participate: having given written informed consent, being involved in face-to-face nursing and medical consultations conducted in Catalan or Spanish, and being of legal age. The exclusion criteria were those people who could not speak Catalan or Spanish fluently or who did not give consent to be audio recorded.

### Sample Size

Despite not having reached the sample calculation established for the general evaluation (800 visits), the protocol provided for a smaller sample to analyze the time savings (120 consultations per group). It has been considered appropriate to publish the results obtained, given the potential positive impact that the deployment of this digital tool can have in the clinical practice of primary care.

### Variables and Data Collection

Sociodemographic information was collected from all participants at baseline using a brief standardized questionnaire administered alongside the study measures. The data collection process is explained in more detail in the previously published protocol [9], but, in summary, it was as follows:

1. The patient attended the consulting room where the project was explained to them, they were invited to participate, and any questions they might have were answered. If they agreed to participate, they signed the informed consent and were assigned alternately to the intervention group or the control group. In the control group, the doctor carried out the consultation in accordance with standard practice. In contrast, in the intervention group, the consultation was recorded with the AI tool and the doctor did not write any notes during the visit but rather reviewed and edited the automatically generated notes once the consultation was complete.
2. The documentation and consultation time were calculated in the group where the tool was used and in the group where it was not.
3. Satisfaction surveys were completed by patients at the end of the consultation (both for the group where the tool was used and for the group where it was not used). The surveys were collected on paper by professionals anonymously.
4. Quality surveys were conducted at the end of each consultation by health professionals. They were carried out directly on a digital platform of the same AI tool used in the consultations.

To calculate the documentation and consultation time in the study, different variables were used depending on the group. In the intervention group, the main variable was the time the professional spent reviewing and correcting the notes automatically generated by the AI tool, measured through the digital platform using records of the start and end of the review. In contrast, in the control group, the time that the professional spent manually writing in the EHRs during the consultation was measured, based on the subsequent analysis of the voice recordings. These two variables, obtained independently for each consultation, allowed us to quantitatively compare the time spent on clinical documentation in both scenarios, with the aim of assessing the potential time savings associated with the use of the tool.

The variables used to evaluate patient satisfaction in both the intervention and control groups were on a Likert scale from 1 (representing the worst score) to 5 (representing the highest score):

- Satisfaction with the professional’s presentation
- Satisfaction with the time allocated by the professional
- Satisfaction with the professional’s willingness to listen to the patient
- Satisfaction with the professional’s willingness to address the person’s health concerns
- Feeling of being in safe hands
- Rating of the treatment and kindness of the professional
- Feeling of the professional’s relaxation level
- Satisfaction with the information received from the professional about their condition
- Rating of feeling cared for and respected regarding data confidentiality
- Detailed explanation of the research by the professional

The variables used to evaluate the satisfaction of professionals with the AI tool were on a Likert scale from 1 (the worst rating) to a maximum of 10 (the highest rating):

- Audio quality
- Transcription quality
- Quality of the medical history field
- Quality of other fields

### AI System and Technical Setup

The AI-assisted documentation pipeline combined ASR and LLM-based postprocessing. ASR was performed using OpenAI Whisper large-v3 (self-hosted), and note structuring was performed using Anthropic Claude 3.5 Haiku (claude-3‐5-haiku-20241022) via Amazon Bedrock with EU-based cross-region inference. Prompt templates and output constraints were defined prior to study initiation based on clinician feedback and internal real-world consultation examples and remained unchanged throughout the study period. End-to-end pipeline latency (from audio upload to draft note availability) was automatically logged and occurred asynchronously. Data were processed within secure, General Data Protection Regulation (GDPR)-compliant cloud environments, and all AI-generated notes were reviewed and approved by the clinician before final documentation.

### Documentation and Consultation Time

In the intervention group, a review button was set up that allowed the time elapsed from the start to the end of the review process to be recorded for each note. Thus, for each audio, the duration of the original consultation, the processing time, and the review time carried out by the health care professional were available. In order to prevent spurious values, reviews that met the following criteria were excluded from the analysis: (1) queries lasting less than 2 minutes, (2) revisions lasting less than 10 seconds or less than 2% of the consultation time, and (3) revisions that exceeded 50% of the consultation time, suggesting incorrect use of the review interface.

Both processing and review times were expressed relative to the total consultation duration (original audio length) to allow comparability across visits. Processing time captured the runtime of the AI documentation pipeline, while review time reflected the clinician effort required to finalize the note.

In the control group, documentation time was estimated by manual review of the consultation audio recordings by a single investigator, who identified documentation segments based on audible keyboard sounds and summed start/end time stamps; recordings were excluded when documentation time could not be reliably determined (eg, poor audio quality, absence of keyboard sounds, substantial background noise or interruptions, or recording failures such as the record button not being pressed or the device being inadvertently left running).

### Accuracy of Notes

As a complementary metric to evaluate the accuracy of the generated notes, the Levenshtein distance between the original transcription and the professionally corrected version was calculated, in absolute terms (number of characters that differed). This measure indicates the minimum number of elementary operations (insertions, deletions, or replacement of characters) necessary to convert one text string into another and is commonly used to quantify the discrepancy between texts [10]. Assuming an average of 6 characters per word and normalizing by the total audio time (in minutes), the proportion of words corrected per minute of consultation was obtained. These data allowed us to objectively quantify the adjustment of automatic transcription and the associated review burden. In addition, clinician feedback was qualitatively coded using a 5-category error taxonomy: (1) omissions, (2) unsupported additions or hallucinations, (3) terminology or numeric errors, (4) context or meaning errors, and (5) style or format issues. Operational or usability comments were excluded. When a single comment referred to multiple error types, each error mention was coded separately to enable a normalized distribution of error mentions across categories.

### Statistical Analysis

The statistical analysis of the study focused on evaluating the 2 main hypotheses: the perceived improvement in the quality of care by patients and professionals and the time saved in writing clinical notes. First, the distribution of the variables was checked. For the descriptive analysis, absolute frequencies were used for categorical variables and means with SD or median with the corresponding quartiles for quantitative variables, according to their distribution. For the comparison of 2 independent samples, the nonparametric Mann-Whitney *U* test or the Student *t* test was applied, depending on the distribution of the variables.

To calculate time savings, 2 aspects were measured separately: the relative time (as a percentage) that the professional had spent on writing notes during the consultation without using an AI-based solution and the relative time that the professional spent on reviewing the notes generated by the tool. For comparison, the nonparametric Mann-Whitney *U* test or the Student *t* test was applied, depending on the distribution of the variables.

All analyses were performed with R software (version 4.0.3; R Foundation for Statistical Computing) [11], considering a confidence level of 95% and a statistical power of 80%.

### Ethical Considerations

The study was conducted in accordance with the ethical principles of the Declaration of Helsinki and complied with applicable regulations for research involving human participants, including those that involved recording patient interactions and subsequent analysis. All participants provided written informed consent prior to their inclusion in the study. Participation was entirely voluntary. No monetary or material compensation was provided to participants. To protect privacy and confidentiality, all recordings and study data were deidentified at source and handled in accordance with applicable data protection regulations. The study was approved by the University Institute for Primary Care Research Jordi Gol Health Care Ethics Committee (Code 3/286 P).

## Results

### Planned Vs Achieved Sample Size

The planned sample size was not fully achieved. The main reason was the reduced number of analyzable observations due to incomplete questionnaire completion and data quality constraints (eg, unusable recordings) and particularly, toward the end of the study period, reduced recruitment intensity in routine practice as clinical workload increased. Therefore, results should be interpreted with caution, and nonsignificant findings should not be interpreted as evidence of no difference.

### Sample Description

**Figure 1.**
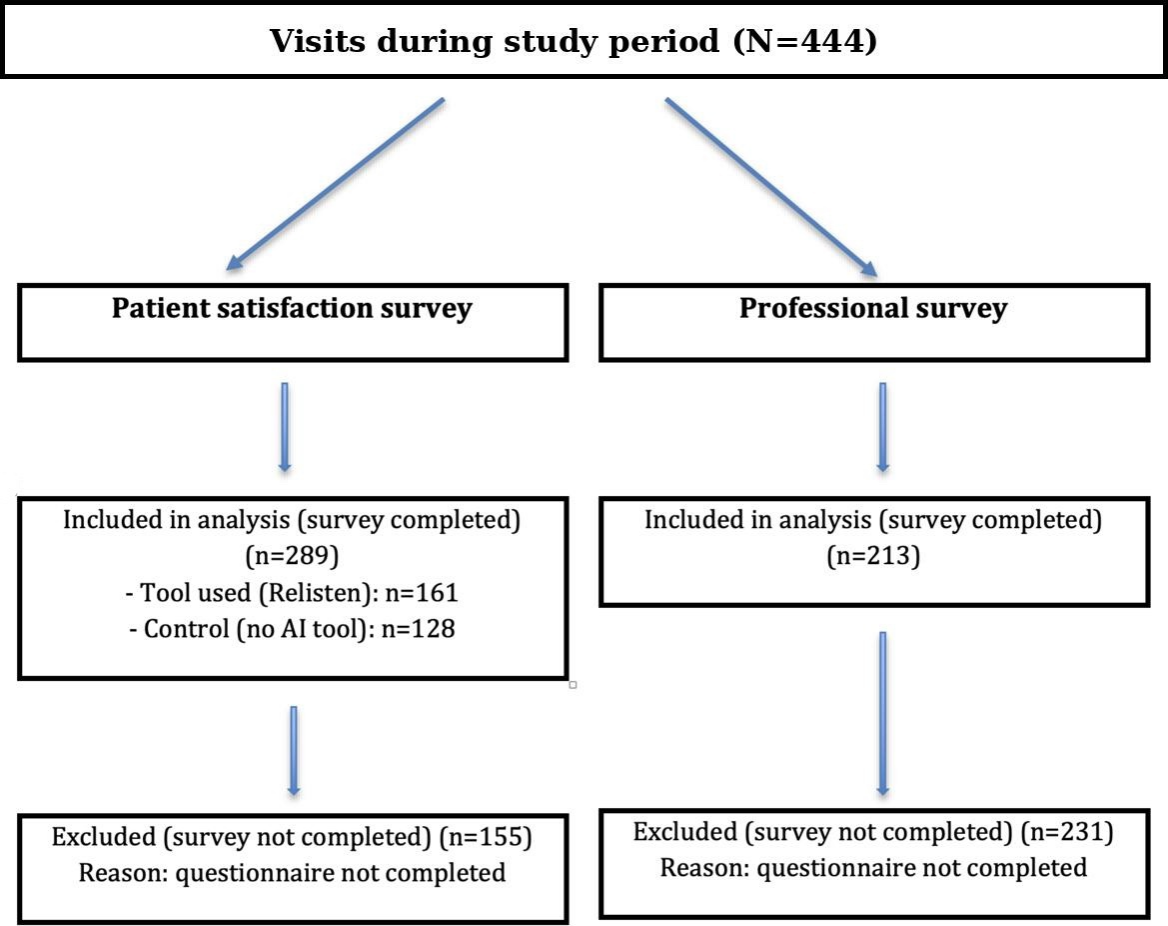
CONSORT (Consolidated Standards of Reporting Trials)-style flow of visits and questionnaire completion. AI: artificial intelligence.

During the study period, 444 visits were made, with a response rate of 65.1% (n=289) in the patient satisfaction surveys and 48% (n=213) in those of professionals, given that not all participants completed the questionnaires. Figure 1 summarizes the flow of visits and survey completion and indicates that the primary reason for exclusion from each analysis was noncompletion of the corresponding questionnaire.

Table 1 shows the description of the sociodemographic variables of the 289 patients who participated in the study and answered the surveys. One hundred sixty-one patients belong to the group that used the tool and 128 to the control group (who did not use the AI tool). No statistically significant differences were observed in terms of age, level of education, and profession, and therefore, there was a homogeneous distribution of the sample in both groups. Women were the majority gender (n=88, 55.3%).

**Table 1.** Description of sociodemographic variables according to control or test.

	Control (n=128), n (%)	Test (n=161), n (%)	*P* value^a^
Age (year)			.72
18‐24	12 (9.38)	13 (8.07)	
25‐34	18 (14.1)	18 (11.2)	
35‐44	13 (10.2)	19 (11.8)	
45‐54	26 (20.3)	24 (14.9)	
55‐69	32 (25)	47 (29.2)	
≥70	27 (21.1)	40 (24.8)	
Gender^b^ (n=287)			>.99
Male	57 (44.5)	71 (44.7)	
Female	71 (55.5)	88 (55.3)	
Educational background^b^ (n=288)			.65
Compulsory education	62 (48.4)	66 (41.2)	
Postcompulsory education	53 (41.4)	77 (48.1)	
I prefer not to say	1 (0.78)	2 (1.25)	
No education	12 (9.38)	15 (9.38)	
Occupation^b^ (n=286)			.91
Full-time worker	63 (49.2)	75 (47.5)	
Part-time worker	11 (8.59)	12 (7.59)	
Student	2 (1.56)	5 (3.16)	
Retired	40 (31.2)	49 (31)	
Other	6 (4.69)	6 (3.8)	
Not working/unemployed	6 (4.69)	11 (6.96)	

a *X*2 test.

b Variables with missing data.

### Patient Satisfaction

**Figure 2.**
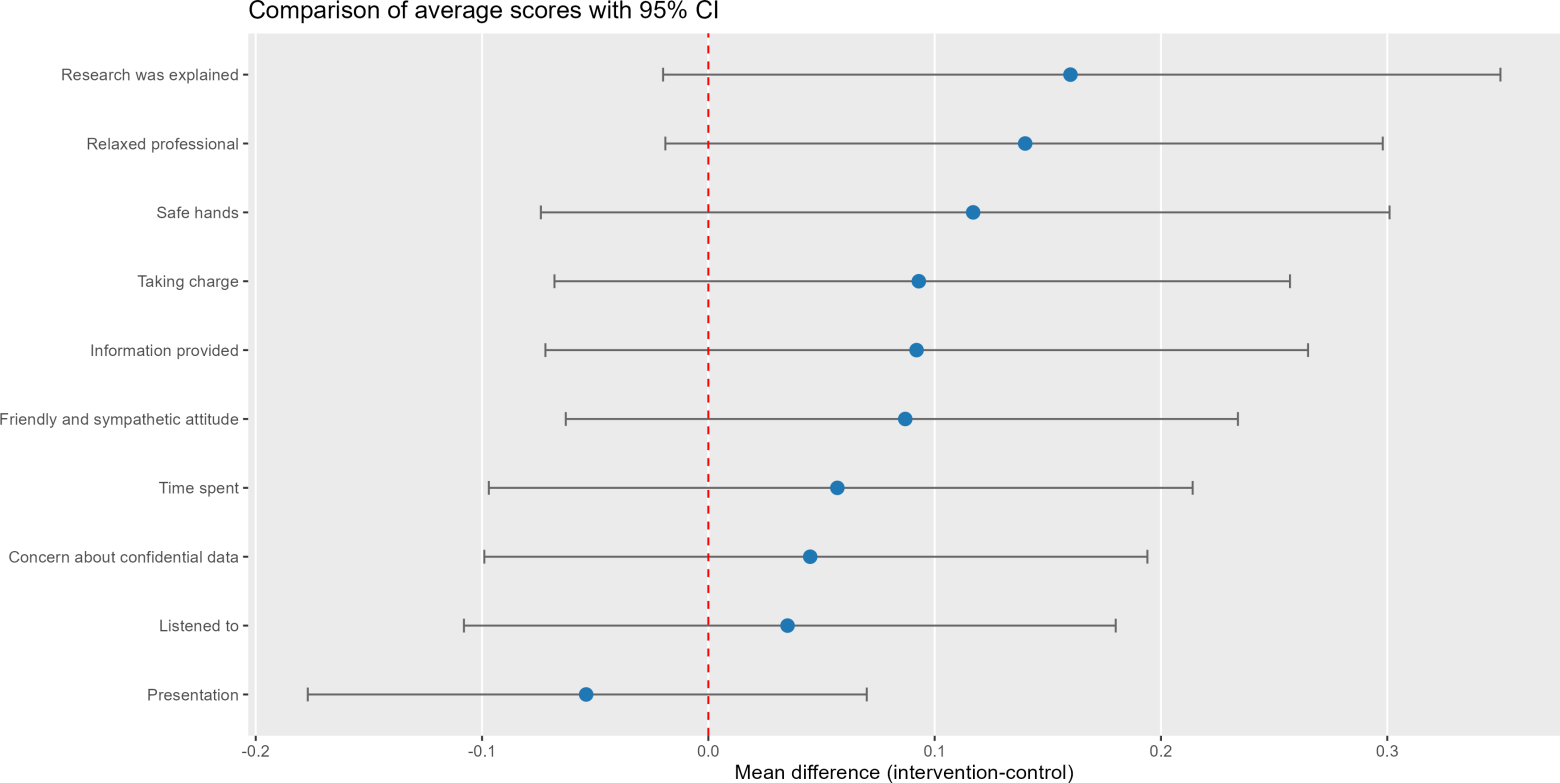
Average score comparison between control and intervention groups.

An analysis was carried out to compare the average of the ratings of the group in which the tool was used with respect to the control group (Figure 2). No statistically significant differences were observed between the rating averages between the two groups (Table 2), with most scores being very high (range 4.35‐4.70).

**Table 2.** Comparison of average scores between control and intervention groups.

Measurement	Intervention, mean (SD)	Control, mean (SD)	*P* value^a^	Mean difference
Presentation	4.65 (0.52)	4.70 (0.53)	.28	−0.054 (−0.177 to 0.070)
Time spent	4.57 (0.60)	4.51 (0.71)	.70	0.057 (−0.097 to 0.214)
Listened to	4.61 (0.55)	4.58 (0.66)	.94	0.035 (−0.108 to 0.180)
Taking charge	4.51 (0.66)	4.42 (0.72)	.28	0.093 (−0.068 to 0.257)
Safe hands	4.51 (0.73)	4.39 (0.81)	.28	0.117 (−0.074 to 0.301)
Friendly and sympathetic attitude	4.61 (0.57)	4.53 (0.72)	.49	0.087 (−0.063 to 0.234)
Relaxed professional	4.58 (0.62)	4.44 (0.75)	.13	0.140 (−0.019 to 0.298)
Information provided by the professional	4.47 (0.67)	4.38 (0.75)	.32	0.092 (−0.072 to 0.265)
Concern about confidential data	4.57 (0.59)	4.52 (0.66)	.82	0.045 (−0.099 to 0.194)
Research was explained	4.51 (0.70)	4.35 (0.85)	.21	0.160 (−0.020 to 0.350)

a Mann-Whitney test.

### Satisfaction of Professionals

A total of 213 ratings were obtained from professionals, 196 from family medicine professionals, and 17 from nurses (Table 3).

**Table 3.** Health professionals’ rating.

	Audio quality, n; mean (SD)	Transcription quality, n; mean (SD)	Current disease, n; mean (SD)	Other fields, n; mean (SD)
Family medicine (n=196)	112; 9.06 (1.18)	112; 8.14 (1.74)	140; 8.05 (1.68)	139; 7.81 (1.76)
Nursing (n=17)	16; 7.62 (1.58)	16; 6.93 (1.52)	17; 7.70 (2.02)	17; 7.11 (1.61)
Total	128; 8.88 (1.31)	128; 7.99 (1.76)	157; 8.01 (1.72)	156; 7.73 (1.76)

Regarding the rating of audio quality, an average score of 8.88 (SD 1.31) was observed. Medical professionals provided slightly higher ratings than nurses; given the marked imbalance in sample sizes, no formal statistical comparison between professional groups was performed.

The rating of the audio transcription obtained a mean overall score of 7.99 (SD 1.76). Likewise, ratings were descriptively higher among medical professionals than among nurses, but no formal between-group comparison was undertaken.

In the rating of the field where the patient’s history was explained, it was rated with an average score of 8.01 (SD 1.72); no formal statistical comparison was performed.

In the rating of the content of the other fields, an average score of 7.73 (SD 1.76) was obtained. Again, results are presented descriptively by professional group without formal statistical testing.

### Documentation and Consultation Time

**Figure 3.**
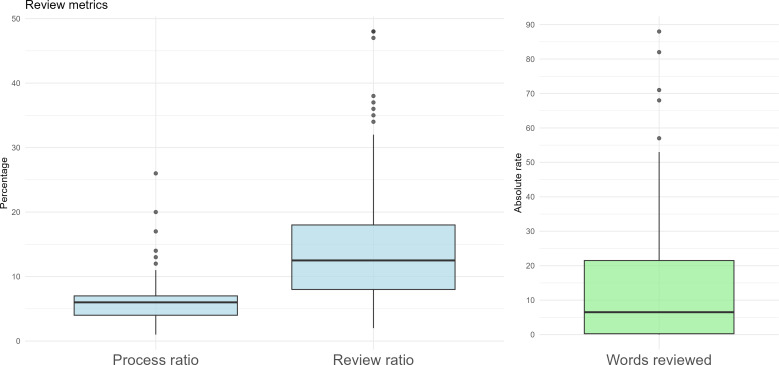
Boxplot of review metrics.

The results of the intervention group are represented in the form of a boxplot (Figure 3), showing the percentage of time spent on each stage with respect to the total duration of the consultation. The average end-to-end processing time (pipeline latency, from audio upload to draft note availability) was 6.63% (SD 4.21), while the revision time accounted for 15.2% (SD 11.23).

Regarding consultations without use of the AI tool (control group), the review of keyboard-usage time did not allow robust conclusions because more than 80% of audio recordings were discarded, mainly due to incomplete recordings in which the consultation was not fully captured. Among the remaining recordings, keyboard usage varied widely (24%‐46%), which precluded reliable quantification of documentation time in the control group. Therefore, supported by both the literature and our sample, an overall estimate of 30%‐35% keyboard use during the consultation was used for comparison. This literature-informed benchmark should be interpreted cautiously, as it represents a broad estimate of documentation-related EHR activity and is not directly equivalent to the intervention metric, which captures only review or editing of the AI-generated note. As for the intervention group, considering that the processing time does not interfere with the care activity and therefore cannot be considered lost time (it was an asynchronous technological process during which the professional could continue to perform other tasks), we present the following calculations as hypothetical, literature-based scenarios intended to contextualize potential savings rather than as empirically measured time savings against the control group. The time savings attributable to the use of the AI tool was quantified as follows:

Savings relative to total consultation time (hypothetical, literature-based): t_savings_total = t_writing_lit − t_review = [30%‐35%] − 15.2% = 14.8%-19.8%

Savings relative to time spent exclusively on documentation (hypothetical, literature-based): t_savings_doc = (t_writing_lit − t_review)/t_writing_lit = ([30%-35%] − 15.2%)/[30%‐35%] = 49.3%‐56.6%

where t_writing_lit is the literature-informed estimate of documentation-related EHR time and t_review is the observed note review or editing time in the intervention group.

These data indicated that using Relisten saved more than half of the time originally used for writing notes.

Even in the conservative assumption that processing time was considered within the calculation, the savings were in an estimated range of 9% to 15% of the total consultation time.

Regarding the estimated revision load derived from the Levenshtein distance, the results obtained are shown in Figure 3. Seventy-five percent of samples presented a low revision load, with less than 24 words modified per minute of consultation, and only 5 cases with higher values, above 48 words per minute, were recorded. In addition, 26% of notes had zero edits (Levenshtein distance=0), indicating that more than one-quarter of drafts were directly usable without manual rework. These categories were defined pragmatically to distinguish low, moderate, and high clinician editing effort and were intended to facilitate interpretability rather than to represent validated linguistic or clinical cutoffs. These results indicate that in most cases, the revision was mild and not very intrusive.

Complementarily, clinician feedback on the generated notes was coded into 5 error categories (excluding operational or usability issues): context or meaning errors were the most frequent (38.7%), followed by terminology or numeric errors (19.4%), omissions (15.6%), style or format issues (8.1%), and unsupported additions or hallucinations (7.5%).

## Discussion

### Principal Findings

The aim of this pilot test was to evaluate the feasibility of implementing an AI tool through the perceived quality of care, the evaluation of the reduction in workload, and freeing up the health care professional from administrative tasks. No statistically significant differences in patient satisfaction were identified, although the average of all scores was very high. This lack of a statistically significant difference may indicate either the absence of a true effect or insufficient statistical power to detect small-to-moderate differences in this study. Professionals positively assessed the quality of the audio and transcription, with a low correction load, which reinforces the operational accuracy of the solution. However, comparisons between professional groups (family medicine vs nursing) are presented descriptively only, given the small number of nurse ratings. Due to the loss of documentation time data in the control group, the impact of the AI tool on documentation time could not be directly measured in a comparative manner within this study.

Although the results obtained regarding patient satisfaction did not show statistically significant differences in any of the dimensions evaluated between the intervention group, which used the AI solution, and the control group, it is worth noting that the average scores in both groups were very high in all categories, ranging between 4 and 5 points out of 5. These results indicate a high overall satisfaction of patients with the care received both with and without the use of the tool. The failure to achieve the sample size no doubt influenced the nonsignificance of the results. There is still little literature on this topic; a study by Cordero et al [12] with surveys sent to 12,000 patients after the implementation of an AI system for note generation in primary care showed a positive perception and a better experience of the visit by patients when the AI tool is used.

Despite having to discard a considerable proportion of visits from the control group for methodological reasons, documentation time data were only available for the intervention group, and no direct comparison of the documentation time between groups was performed. Several studies place the percentage of consultation time spent on clinical documentation in the EHR between 24% and 54% [13-18]. The study by Pérez-Santonja et al [19], carried out in the field of Spanish primary care, which estimated this percentage at 38.33%, was particularly noteworthy. These literature findings provide context regarding the magnitude of documentation workload in routine primary care practice, while the present study contributes empirical observations on documentation and review time in consultations supported by an AI-based transcription tool. In addition, saving time in documenting the visit can free up minutes that can be reallocated to active listening and health education, key aspects in primary care. However, most studies use pre-post designs without randomization or convenience samples, so they are in line with the recommendations of Duggan et al [15]. Multicenter randomized controlled trials will be necessary to consolidate the evidence.

The estimated revision load derived from the Levenshtein distance indicates that, in most cases, professional intervention after using the AI tool was minimal, with fewer than 24 corrected words per minute of consultation and 26% of drafts requiring no edits. These results point to a good quality initial transcription, which significantly reduces the time and effort required to produce the final clinical note. However, Zhou et al [20] analyzed the error rate before and after editing, showing that the error rate in the version generated by the voice software was 7.4%, 0.4% after the transcriptionist’s review, and 0.3% in the final version signed by the professional. Complementing these findings, our qualitative error analysis shows that context and meaning errors were the most prevalent, reflecting the difficulty of accurately interpreting and structuring multiproblem clinical encounters. By contrast, style and format issues were infrequent, likely influenced by reviewer participation bias, as clinicians tend to report clinically relevant inaccuracies rather than purely stylistic concerns, while unsupported additions or hallucinations—less affected by this bias—remained the least frequent error category, reflecting the system’s strong emphasis on avoiding fabricated clinical information.

In this regard, it must be emphasized that although these tools have the potential to improve workflow efficiency, manual editing and review by health care professionals remain necessary. Therefore, these results support the use of such tools as assistive systems for clinicians and under no circumstances as a complete replacement for professional judgment and responsibility.

### Ethical Implications

Beyond technical performance, the use of LLM-enabled documentation tools in routine care raises ethical and regulatory questions that are increasingly emphasized in recent literature. Key concerns include (1) patient safety (eg, the risk of incorrect or misleading text in clinical records), (2) potential bias that could differentially affect patients or clinicians, (3) limited transparency of model behavior, and (4) questions of accountability when AI-assisted notes influence downstream clinical actions or administrative decisions [21,22]. Issues around privacy, data governance, and oversight are also central, particularly when tools rely on recorded clinical encounters and generate content that becomes part of the medical record [21].

In this proof-of-concept study, these risks are partially mitigated by the intended workflow: the system is used to support documentation, and the generated note is reviewed and approved by the clinician before being incorporated into the EHRs.

### Implications for Clinical Practice

The implications for clinical practice in primary care are focused on the finding that improving and reducing the documentation collected by professionals can translate into more available appointments and reducing the workload, especially in administration, for primary care professionals, which has been widely documented in other research [13,23,24]. However, real-world implementation requires attention to practical barriers, including costs associated with deployment and maintenance, time and resources needed for staff training and onboarding, and technical work required to integrate the tool within existing EHR infrastructure and the professional’s routine day-to-day workflow. Finally, scalability across centers will depend on interoperability with different local systems, alignment with governance and data-protection requirements, and ongoing monitoring once deployed to ensure that the AI tool’s performance remains stable across settings and over time.

This proof of concept was conducted in the Catalan primary care context, and its findings should be interpreted accordingly. While documentation burden is a common issue across health systems, transferability may be influenced by differences in EHR interoperability, local documentation practices, and governance requirements for recording and processing clinical encounters. Therefore, generalization beyond this setting should be supported by context-specific evaluation.

### Limitations

This study has several important limitations. First, limitations related to sample size and statistical inference must be considered. The target sample size was not reached; however, given the feasibility focus and proof-of-concept design, the achieved sample was considered sufficient for descriptive evaluation. With the achieved sample sizes (control n=128 and intervention n=161) and assuming 2-sided *α*=.05 (SD 1) on the 1‐5 Likert scale, the minimum detectable difference with 80% power was approximately 0.33 Likert points. Therefore, smaller effects may not have been detectable, and nonsignificant findings should be interpreted with caution. In addition, we did not apply ordinal regression models, compute standardized effect sizes, or adjust for multiple comparisons; therefore, inferential findings should be interpreted as exploratory. Finally, patient satisfaction showed a likely ceiling effect (most means >4.5/5), which may have reduced sensitivity to detect subtle changes in the patient-clinician interaction.

Second, limitations affecting internal validity and generalizability include the allocation method, clustering, and missingness. Participants were assigned to the intervention or control groups in an alternating manner as they entered the study. This method does not constitute true randomization, as it is predictable and can result in selection biases if not adequately controlled. This lack of randomness may have compromised the internal validity of the results. For further studies, randomized controlled trials would be necessary to validate their efficacy. In addition, potential clustering effects (eg, by primary care center and clinician) were not formally modeled; therefore, SEs and CIs may be underestimated due to within-center correlations. Surveys were collected anonymously and were not linkable to EHR or administrative data for nonresponders; therefore, we were unable to compare respondents with nonrespondents and cannot rule out nonresponse bias. Furthermore, the sample difference between professional categories (medicine and nursing) must be considered when interpreting differences in satisfaction ratings.

Third, several limitations relate to measurement of transcription quality, workflow burden, and operational performance. Standard ASR metrics such as word error rate (WER) could not be computed, as no independent ground-truth transcriptions of the consultation audio recordings were available. Operational performance indicators were only partially captured in this proof-of-concept study. While model versions and end-to-end latency were documented and clinician editing burden was assessed (including the proportion of drafts requiring no edits), failure modes (eg, recording or pipeline errors), retry rates, and standardized system telemetry were not prospectively instrumented. Future work should include comprehensive logging to enable technical benchmarking and scalability assessment. In addition, a “zero-edit” draft (Levenshtein distance=0) reflects textual agreement but does not guarantee clinical correctness and may be affected by automation bias (eg, reduced vigilance under workload); therefore, all AI-generated notes must be reviewed and approved by a clinician before being stored in the health record. Moreover, we could not stratify performance by language (Catalan vs Spanish) because many consultations involved code-switching between both languages, and data were collected anonymously, preventing reliable attribution of metrics to a single language.

Fourth, limitations specific to documentation-time estimation and benchmarking must be acknowledged. The loss of data on documentation time in the control group prevented a direct comparison with the intervention group regarding efficiency in writing clinical notes; time-related findings are therefore exploratory and should be interpreted with caution. Future studies should use a more robust design (eg, a pre/post or randomized controlled trial with high-quality time measurements in both groups) to accurately quantify the impact on documentation time. In addition, our “note review time” metric is not directly comparable to literature estimates of total EHR documentation time, which typically include broader EHR activities (eg, chart navigation, order entry, and reviewing prior history). Therefore, any comparison with published benchmarks should be interpreted cautiously and, at most, as an upper-bound estimate, as the AI tool does not remove the nontranscription EHR tasks included in those estimates. Furthermore, the literature benchmark used (2016-2018) [13,14] may introduce a temporal mismatch, as EHR workflows and administrative demands may have changed by 2024/2025; therefore, this historical comparator should be interpreted cautiously.

Finally, potential conflicts of interest and analytic independence should be considered. Data custody and analysis were not fully independent from the tool developer, which may introduce residual bias; future studies should include independent analysis.

### Conclusions

The implementation of an AI tool for the automatic writing of clinical annotations in primary care in Catalonia was feasible and showed a documentation workflow compatible with routine clinical practice, high professional acceptance, and no negative impact on patient satisfaction. The results suggest the possible use of these tools as a form of support for health care professionals. Although this study does not allow conclusions to be drawn regarding comparative or quantified time savings, the findings provide empirical evidence on documentation and review processes associated with AI-based transcription tools. Despite the sample and important methodological limitations, the results support conducting randomized clinical trials that would confirm the benefits for health care quality and system efficiency.

## Abbreviations

AI: artificial intelligence
ASR: automatic speech recognition
CAP: primary care center
EHRs: electronic health records
LLMs: large language models
